# Indirect Inguinal Hernia Containing a Fallopian Tube and Ovary in a Reproductive Aged Woman

**DOI:** 10.1155/2014/437340

**Published:** 2014-06-16

**Authors:** Ashley Graul, Emily Ko

**Affiliations:** ^1^OB/GYN, Pennsylvania Hospital, Philadelphia, PA 19107, USA; ^2^Gynecologic Oncology, University of Pennsylvania, Philadelphia, PA 19104, USA

## Abstract

An indirect inguinal hernia containing an incarcerated fallopian tube and ovary is extremely rare in adult females. The current report describes a woman of reproductive years presenting with an irreducible indirect hernia which required the surgical intervention of a general surgeon as well as counseling regarding future fertility by a gynecologist. The diagnosis was made by physical and sonographic examination and was confirmed by CT scan and surgical intervention. We suggest a multimodel and multidisciplinary approach in order to safely and efficiently preserve ovarian and fertility function in young women who present with an inguinal hernia containing reproductive organs.

## 1. Introduction

Inguinal hernias occur in less than 5% of women [1]. Even though infrequent, when present, hernias must be evaluated urgently due to possible incarceration (trapped organs) or strangulation (disruption of blood flow) of organs, including, on rare occasion, the ovary and fallopian tube. In most circumstances, the diagnosis of an inguinal hernia can be made based on history and physical exam alone. Inguinal hernias may present as an asymptomatic finding such as a painless bulge in the groin area, or in a subacute or acute manner, with mild to severe abdominal-pelvic pain [2]. When an adult woman presents with severe abdominal pelvic pain, abdominal wall hernias must be considered in the differential. Hernias may frequently be overlooked particularly if not assessed in a physical exam.

These hernias may contain a variety of visceral organs, of which intestines or omentum is most commonly found, resulting in an incarceration or strangulation which would be a surgical emergency. The ovary or fallopian tube may also become entrapped, although this is rarely considered. The patient may present in a nondescript manner and describe a heaviness or dull discomfort in the groin that is most pronounced when intra-abdominal pressure is increased. Typically, hernias can be diagnosed by careful palpation on physical exam if it is considered and confirmed by ultrasound imaging. However, when ultrasound results remain ambiguous and there is concern for entrapped organs, a CT scan can be performed to provide definitive diagnosis and aid in proper counseling, consultation, and timely decision making for urgent surgical management in order to avoid organ necrosis.

We present a rare case of a woman with an indirect, nonreducible inguinal hernia containing a normal appearing ovary and fallopian tube which required urgent surgical intervention to prevent strangulation of the adnexa and to ensure fertility preservation.

## 2. Case Report

A 25-year-old G2P1011 presented to the Emergency Department with increasing left groin pain that radiated to the mons for the past five days. The patient noticed a small bulge in the groin area over the past five months but denied pain previously. Over the past five days, however, the patient reported having immediate pain after valsalva, for example, after each bowel movement. She denied any urinary symptoms, nausea, or vomiting. She had no significant past medical history or surgical history.

Physical exam revealed a 2 cm × 2 cm mass over the left mons abutting the pubic symphysis. The mass was tender to palpation. The abdomen was soft, nontender, and without peritoneal signs. Vital signs were BP 119/67, HR 94, Temp 98.3, and RR 16. Lab studies including chemistry panel, complete blood count, and urine pregnancy test were within normal limits.

**Figure 1:**
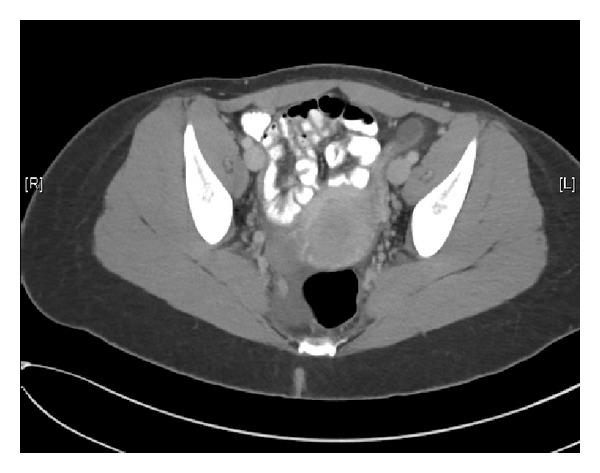
CT scan.

**Figure 2:**
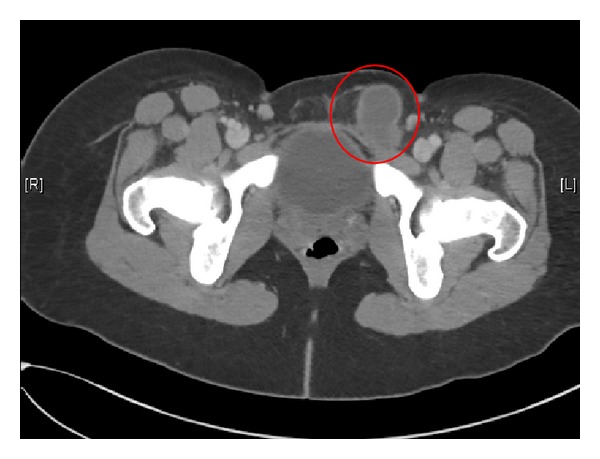
CT scan.

The patient was initially evaluated by emergency room physicians, and an ultrasound was ordered to further determine the contents of the inguinal mass. The ultrasound report findings suggested “thick walled, complex tubular fluid collection abutting and inseparable from the left ovary and extending into the left groin, possibly representing a dilated and herniated fallopian tube.” Given the concern for possible entrapped organs, a CT scan was ordered, and consultation requests for both General Surgery and OB/GYN were obtained (Figure 1). The CT findings included “left inguinal hernia containing fluid-filled, tubular structure, possibly a dilated left fallopian tube. Adjacent fluid and stranding, ischemic change cannot be excluded.” (Figure 2).

A bedside hernia reduction was attempted but was unsuccessful. The patient was evaluated by OB/GYN and counseled regarding the possibility of unilateral salpingo-oophorectomy if the adnexa appeared to be necrotic. The patient was taken for exploration and she underwent an open left inguinal hernia repair with mesh placement. Examination revealed an indirect hernia containing an incarcerated, dilated fallopian tube with a healthy, viable ovary sitting within the hernia sac. The contents were easily reduced back into peritoneal cavity without difficulty. The inguinal floor was reinforced with Prolene mesh.

## 3. Discussion

Inguinal hernias consist of direct hernias (defined by protrusion through Heselbachs triangle, medial to the inferior epigastric vessels) and indirect hernias (defined by protrusion through the inguinal ring, lateral to the inferior epigastric vessels) [2]. In adult women, indirect hernias are more common than direct hernias and typically occur during age 40–60 [1]. Most of these hernias contain intestinal contents and rarely viscera such as female adnexa (ovaries or fallopian tubes) in 3% of hernia cases [3]. If entrapment of the female adnexa occurs, it is found more frequently in infants due to anatomical causes including (1) a relatively short inguinal canal, (2) a canal that has an oblique direction through the abdominal wall, and (3) a diverticulum of Nuck, a peritoneal pocket associated with the round ligament that corresponds to the vaginal processus in the male infant [3–6].

In contrast, the occurrence of entrapped adnexa within an indirect hernia in women, as seen in our patient, would be unexpected and uncommon. When discovered in adult females, the majority of hernias reported are found in perimenopausal or postmenopausal women [3, 7]. However, the differential must be considered in premenopausal women as well, as with the patient presented. Additionally, in contrast to the present report, previous case reports of fertile women with entrapped adnexa have been associated with abnormalities of the fallopian tube, such as paratubal cysts and hemorrhagic cysts of the ovary which may cause a weighted descent of the organ and predisposition for entrapment [7, 8]. Further risk factors may include lengthening of the broad, uterine, or ovarian suspensory ligaments in high parity patients resulting in displacement of adnexal structures and high intra-abdominal pressures from frequent valsalva maneuvers in patients with chronic cough or frequent heavy lifting [5]. Our patient, however, had no adnexal enlargement or pathology identified, nor any of these risk factors listed.

Timely management must be undertaken to ensure prompt surgical intervention to reduce the risk of ovarian damage and subsequent infertility. This will likely require collaborative efforts by gynecologists and general surgeons and recognition of this condition by both parties in order to ensure preservation of fertility. In past reports where female adnexa were involved, more than half required oophorectomy secondary to strangulation (*n* = 4/7) [3]. When extending to cases involving female infants or children, 27% (*n* = 4/15) of those who presented with irreducible ovaries were found to have infarcted ovaries at surgery [6] as reported in a series of 1699 children with inguinal hernia. Incarceration of adnexa alone may decrease the blood supply, and torsion (which is not infrequently found with enlarged adnexa) would likely compromise blood flow further. Thus, female adnexa are particularly vulnerable to damage when entrapped in inguinal hernias, and failure to recognize this may result in an infarcted and unsalvageable ovary and/or fallopian tube.

## 4. Conclusion

In summary, we have described the rarity of a normal fallopian tube and ovary within an indirect inguinal hernia in a premenopausal adult female. Furthermore, diagnosis of entrapped viscera including the adnexa must be considered in the differential diagnosis of hernias in adult women, in order to ensure proper surgical and medical management to ensure preservation of fertility. Surgical intervention in a timely fashion may be necessary in order to prevent and relieve torsion and to return normal perfusion to the adnexa. Multiple imaging studies may be necessary to assist in diagnosis, including ultrasound and/or cross-sectional imaging by computed tomography (CT). By considering a gynecologic etiology when faced with inguinal hernias in women and using a multimodel approach including history, physical examination, imaging, and surgical management, one can safely and efficiently preserve ovarian and fertility function in young women.
